# The necessity for standardization of glioma stem cell culture: a systematic review

**DOI:** 10.1186/s13287-020-01589-8

**Published:** 2020-02-26

**Authors:** Lei Zhang, Hongwei Yu, Yuhui Yuan, John S. Yu, Zhenkun Lou, Yixue Xue, Yunhui Liu

**Affiliations:** 1 Department of Neurosurgery, Shengjing Hospital of China Medical University, # 36 Sanhao Street, Heping District, Shenyang, China; 2 Department of Neurosurgery, Cedars-Sinai Medical Center, Los Angeles, USA; 3 Department of Oncology, Mayo Clinic, Rochester, USA; 4 Department of Neurobiology, School of Life Sciences, China Medical University, Shenyang, China

**Keywords:** Glioma stem cell culture, Standardization, Serum-free media, Components

## Abstract

**Background:**

The cancer stem cell hypothesis is an old idea which has been revived in recent years for many cancers, including gliomas. However, this concept has become controversial due to a series of studies with conflicting results.

**Methods:**

A systematic literature search was conducted in PubMed and the Web of Science database to analyze studies using serum-free medium and its components in glioma stem cells, glioma stem-like cells, glioma-initiating cells, or glioma neurosphere cultures. All the studies reviewed were published between 1970 and 2019. We found that no standardized culture method was used, and the data were incomparable due to differing culture conditions and the use of media with different components.

**Conclusions:**

Here, we review the most commonly used serum-free media and added components for glioma stem cell culture while highlighting the function of each component used in the media. We emphasize the necessity for standardization of glioma stem cell culture and propose a standard culture medium to prevent bias in glioma stem cell research.

## Background

The concept of cancer stem cells was first proposed about 150 years ago as the “embryonal rest” theory of cancer by Virchow and Cohnheim [1], where a small subpopulation of cells was hypothesized to be responsible for the origin of cancer. More recently, the existence of cancer stem cells was first confirmed by Lapidot and Dick in leukemias [2, 3], followed by Al-Hajj and Clarke in the solid tumors of breast cancer [4] and Singh and Dirks in brain cancers [5, 6], especially in gliomas. Glioma stem cells cultured in epidermal growth factor (EGF) and basic fibroblast growth factor (bFGF) have been reported to more closely mirror the phenotype and genotype of primary tumors than serum-cultured cell lines [7]. However, all research laboratories culture cancer stem cells using their own methods, and they may use different kinds of culture media and different additives. This may lead to discrepancies in the results between laboratories and may be one of the reasons why the cancer stem cell theory was questioned [8, 9]. The lack of a standard culture system for glioma stem cells and ununified methods for their generation may affect downstream results. This is also one of the reasons for the debate over CD133 as a marker for glioma stem cells [10]. Therefore, there is a critical need to review the methods, media, and components used for glioma stem cell culture in the literature. Additionally, standardized glioma stem cell culturing methods are necessary to prevent biased results caused by the media components. We searched the PubMed and Web of Science databases using the key words glioma stem cell, glioma stem-like cells, glioma-initiating cells, and glioma neurosphere culturing.

## Serum-free medium

Chemically defined, serum-free media was pioneered by *Sato* et al. [11] to replace serum with the addition of selected hormones, promoting growth and stimulating differentiation of specific cells. Recently, the serum-free media used to culture neural stem cells was modified to be used for glioma stem cell culture. To better understand glioma stem cell culture, it is important to know the components of the media and supplements being used. In general, five kinds of serum-free media are commonly used for glioma stem cell culture:

### Media hormone and salt mixture (media hormone mix)

The media hormone mix was originally used by *Weiss* et al. for neural stem cell culture, and it was composed of a 1:1 mixture of Dulbecco’s modified Eagle’s medium (DMEM) and Ham’s F-12-based medium. DMEM had high amino acid content, and Ham’s F-12 contained a highly enriched mixture of trace elements. This medium consisted of a basal medium and a hormone and salt mixture. The basal medium was DMEM/F-12 with 0.6% glucose, 2 mM glutamine, 3 mM sodium bicarbonate, and 5 mM HEPES. The hormone and salt mixture included insulin (25 μg/ml), transferrin (100 g/ml), progesterone (20 nM), putrescine (60 μM), and selenium chloride (30 nM). The hormone and salt mixture, which contained apo-transferrin, was equivalent to Gibco N2, which contained holo-transferrin [12]. *Dirks* et al. used this media [12, 13] combined with a growth factor cocktail from the Weissman group [14] to generate glioma stem cells. Dirks’s medium was composed of media hormone mix with 1× antibiotic-antimycotics. It was supplemented with 20 ng/ml of bFGF, 20 ng/ml of EGF, 10 ng/ml of leukemia inhibitory factor, 60 μg/ml of *N*-acetylcysteine [14], and 2% of neuronal survival factor-1 (NSF-1) [5]. This medium was widely used for glioma stem cell culturing by the Rich group [15, 16] and other laboratories [17] after Dirks’s publication in *Nature* [6].

### Neurobasal medium with N2 and B27 supplements

Neurobasal medium is a widely used medium for neuronal cultures, originally formulated by *Brewer* et al. to support the survival of rat hippocampal neurons [18, 19]. Its components are similar to DMEM/F-12, but with reductions in NaCl to 3.0 g/liter, cysteine/cystine to 10 pM, and glutamine to 0.5 mM. The total osmolarity with B27 was 235 mOsm, substantially less than the 335 mOsm of DMEM [19]. As the neurobasal medium also includes the biological antioxidants vitamin E, glutathione, pyruvate, catalase, and superoxide dismutase, the neurobasal medium is not recommended for survival studies involving free radical damage and oxidation [19]. The neurobasal medium with N2 and B27 was originally used by the Fine group for glioma stem cell cultures. Fine’s medium consisted of neurobasal media with Gibco N2, B27 (0.5 × each), bFGF (50 ng/ml), and EGF (50 ng/ml) [7]. The glioma stem cells cultured in this medium were reported by the Fine group to be more reliable than many other cancer cell lines [7].

### NeuroCult proliferation medium (STEMCELL Technologies)

NeuroCult proliferation medium may be manufactured according to the Vescovi group’s formula [17]. It was the same media used by the Weiss group and was often used for glioma stem cell research [20]. Vescovi used to work in the Weiss group at the University of Calgary and had published a few studies using this medium [21].

### Neurobasal media with B27 supplements

Neurobasal media with B27 was originally chosen for neural cell culture. Glial growth was shown to be reduced to less than 0.5% of the nearly pure neuronal population using this media. This media with the above formulation not only supported the growth of neurons from embryonic rat striatum, substantia nigra, septum, and cortex, and the growth of neurons from neonatal dentate gyrus and cerebellum, but also supported the growth of other neuron types as well [22]. The formulation was also used to study the effects of growth factors and hormones by the Brewer group [19]. Recently, some researchers have used this media for glioma stem cell culturing and glioma stem cell-related research [23].

### DMEM/Ham’s F-12 with B27 supplements

DMEM/Ham’s F-12 with B27 was initially used for neural stem cell culture [24], and it had also been used recently to culture glioma stem cells [25]. The use of DMEM/Ham’s F-12 without other added reagents such as glucose, glutamine, sodium bicarbonate, and HEPES may have resulted in the discrepancies between their results and those using the Weiss media. Therefore, DMEM/F-12 supplemented with B27 is not well formulated for glioma stem cell culture.

## Supplements

Three kinds of supplements are often used in glioma stem cell culture: N2, hormone and salt mixture, and B27.

### N2 supplement

N2 supplement was first formulated by *Bottenstein* et al. in 1979 as a serum-free synthetic medium supplemented to support the proliferation of neuronal cells. It contained 5 μg/ml crystalline bovine insulin, 100 μg/ml transferrin (90% iron-free), 20 nM progesterone, 30 nM sodium selenium, and 100 μM putrescine dihydrochloride in place of serum and was used to culture rat neuroblastoma cell lines [26, 27]. Recently, the N2 supplement has been widely used for glioma stem cell culture. N2 supplements can be either made from scratch or purchased from Thermo Fisher as Gibco N2. However, the commercially available N2 contains holo-transferrin, which is different from Bottenstein’s original N2 formula. Bottenstein’s N2 contains 90% iron-free apo-transferrin. A previous study showed that holo-transferrin (iron-saturated) could stimulate cell proliferation, but apo-transferrin (iron-deficient) did not [28]. Iron salts and transferrin are specifically required for cell division in cultured 3T6 cells [29, 30]. Therefore, the difference in function between holo-transferrin and apo-transferrin may affect the growth of cultured glioma stem cells.

### Hormone and salt mixture (hormone mix)

*Di Porzio* et al. reported that a virtually pure neuronal population could be obtained by using serum-free medium supplemented with 25 μg/ml insulin, 100 μg/ml transferrin, 2× 10^− 8^ M progesterone, 6 × 10^− 5^ M putrescine, and 3 × 10^− 8^ M selenite salt [31]. *Di Porzio* et al. modified Bottenstein’s N2 formulation by increasing the concentration of insulin from 5 μg/ml to 25 μg/ml and reducing the concentration of putrescine from 100 to 60 μM. The Weiss group chose di Porzio’s medium to generate neural stem cells from mice, and this medium was also called hormone mix in place of serum [13, 32–35]. The hormone mix is similar to Bottenstein’s N2 or Gibco N2, except that the concentrations of insulin and putrescine are different. The Weiss group used the hormone mix with apo-transferrin (iron-deficient) as Bottenstein’s N2 did. Recently, the Weiss group also used this media to culture glioma stem cells [36].

### B27 supplement

B27 supplement was originally developed by *Brewer* et al. in 1993 after the earlier development of N2 by Bottenstein. It contained a total of 20 components with optimized concentrations of the ingredients in Bottenstein’s N2 (insulin, transferrin, progesterone, putrescine, and selenium) along with the thyroid hormone T3, fatty acids, vitamin E, and other antioxidants [22]. *Brewer* et al. reported that basal medium with B27 supplement optimized the survival of primary rat embryonic neurons after 4 days in culture [19, 37]. However, the Weiss group noted that B27 contained retinyl acetate and triiodo-l-thyronine, which may promote the differentiation of various precursor cells [38]. *Noble* et al. also mentioned that the redox state of the precursors might determine their differentiation status [39]. For this reason, some researchers had difficulties with their neuron cultures when using B27 [40]. When used in combination, neurobasal and B27 could allow the long-term growth of primary embryonic hippocampal and other brain neurons [19]. For neurosphere culture, the formulation should consist of neurobasal, 2% B27 minus retinyl acetate, and 0.5 mM GlutaMAX supplemented with 10 ng/ml EGF and 10 ng/ml bFGF (10 ng/ml platelet-derived growth factor-bb was also necessary for mouse neurosphere culture). B27 without retinyl acetate slows differentiation [41]. Another report mentioned that the defined culture medium that promoted neuron regeneration inhibited the growth of human primary glioblastoma [42].

## Growth factors and reagents

### EGF and bFGF

EGF and bFGF are the two essential growth factors which are used for the isolation of neural stem cells (NSCs) from the embryonic and adult brain in vitro. In addition, EGF and bFGF also serve as mitogens for neural stem cells [13, 21, 35, 43]. Recently, EGF and bFGF have been used in glioma stem cell culture as well as for neural stem cell culture.

#### EGF

EGF is a powerful mitogen for numerous non-neuronal cells. The mitogenic and trophic actions of EGF on embryonic and early postnatal cells signify its importance in neuronal development in the central nervous system (CNS) [35]. EGF signaling through the EGF receptor is essential for not only the proliferation of neural stem cells (NSCs) [13, 35, 44] but also the proliferation of glioma stem cells [36].

#### bFGF

bFGF, another mitogenic growth factor, participates in the production of neurons and enhances the proliferation of neuronal precursors from the embryonic rat brain [45]. bFGF has been reported to act chiefly through FGF-receptor-1. However, both receptor-binding affinity and growth factor-mediated mitogenicity in many cell types, including neuroepithelial cells, were dependent on heparin [46]. Therefore, heparin is needed to stabilize the activity of bFGF. The Covarrubias group found that bFGF initially acts as a differentiation factor in the mesencephalon and makes the cells responsive to EGF. In addition, bFGF also promoted the survival of cultured cells [47].

Recently, the Fine group reported that tumors generated by cells from glioblastoma multiforme (GBM) spheres grown in EGF and bFGF were much more similar to the human glioblastoma than tumors initiated by glioma cell lines [7, 48]. In addition, the Dirks group also reported that EGF and bFGF enhanced the survival, proliferation, and sphere size of glioma stem cells [5]. The Weiss group reported that GBM spheres generated in serum-free medium with EGF and bFGF could generate spheres that were able to differentiate into cells that expressed astrocytic, oligodendroglial, and neuronal markers [36]. The Weiss group also reported that the GBM spheres, derived from glioblastoma patients with altered differentiation profile rather than from normal NSCs, could give rise to highly invasive tumors [36].

### Leukemia inhibitory factor

Leukemia inhibitory factor is a cytokine belonging to a family of interleukin 6-related cytokines, and it is originally identified as a factor that inhibits the proliferation of the undifferentiated [49]. In addition, it also prevents differentiation in normal embryonic stem cells [49]. The Weissman group isolated neural stem cells using culture medium consisting of Ex Vivo 15 medium with N2 supplement, EGF, bFGF, leukemia inhibitory factor (an error in the original publication listed this as lymphocyte inhibitory factor instead of leukemia inhibitory factor), neural survival factor-1, and *N*-acetylcysteine [14]. This medium was originally used by the Carpenter group [50] with the following formulation: DMEM/F-12-based medium supplemented with 0.6% glucose, 25 μg/ml insulin, 100 μg/ml transferrin, 20 nM progesterone, 60 μM putrescine dihydrochloride, 30 nM sodium selenite, 2 mM glutamine, 3 mM sodium bicarbonate, and 5 mM *N*-2-hydroxyethylpiperazine-*N*-2 ethane sulfonic acid (HEPES). This medium was also supplemented with 20 ng/ml EGF, 20 ng/ml bFGF, 10 ng/ml leukemia inhibitory factor, and 2 μg/ml heparin [50]. *Carpenter* et al. suggested that the cells could be maintained and expanded in a serum-free medium containing these three factors (EGF, bFGF, and leukemia inhibitory factor). Using all three, the cultured cells could be expanded and remained multipotent for at least 1 year in vitro. Recently, the leukemia inhibitory factor has been reported to have an essential role in the regulation of glioma stem cells in human glioblastoma [51].

### Heparin and heparan sulfate

Heparin and heparan sulfate are glycosaminoglycans that modulate numerous biological processes. Heparin, a mast cell-derived polysaccharide, was originally characterized as an anti-coagulant and mediator of inflammatory responses [52]. In addition, heparin, but not other proteoglycans, had been reported to potentiate the mitogenic effects of bFGF on mesencephalic precursor cells [53]. Heparin can also stabilize bFGF and modulate striatal precursor cell behavior in response to EGF [54]. It can increase the survival of rat EGF-generated CNS precursor cells when the B27-supplemented medium is used [55]. *Svendsen* et al. reported that up to 15-fold expansion of low-density neural stem cells derived from the lateral ventricle wall, the hippocampus, and the spinal cord of adult rats was achieved within 1 week [56].

Heparan sulfate (HS) is a highly sulfated linear polysaccharide. Unlike heparin, heparan sulfate is usually expressed on the cell surface and in the extracellular matrix of all animal cells [57]. Heparan sulfate proteoglycans (HSPG) are obligatory for receptor binding and mitogenic activity of bFGF [52]. *Thompson* et al. reported that the binding of heparin or heparan sulfate to FGF was essential for FGF-mediated signal transduction and mitogenicity [58]. In glioma stem cell culture, both heparin [59] and heparan sulfate [36] had been used to stabilize the activity of bFGF. HSPG have been shown to regulate a wide range of cellular functions and bioprocesses by acting as a co-receptor for bFGF and affecting its bioactivities [60]. This work provided an important step toward the development of standardized protocols for highly efficient in vitro expansion of neural stem cells from the adult central nervous system, moving more closely to the clinical use of neural stem cells as well as of glioma stem cells [36, 61].

### Neuronal survival factor-1 and *N*-acetylcysteine

Neuronal survival factor (NSF)-1 (2%) and *N*-acetylcysteine (NAC) were used by the Weissman group to culture neural stem cells [14]. NSF-1 promotes the survival of neural stem cells. NAC, a mucolytic agent and a thiol-containing antioxidant, has a sulfhydryl group that disrupts the disulfide bonds of glycoproteins in mucus and provides a cysteine source for the intracellular synthesis of glutathione [62]. It may prevent DNA damage by decreasing intracellular reactive oxygen species (ROS) levels, and it can increase cell viability in murine hematopoietic stem/progenitor cells [63]. Recently, these two reagents were also used to generate glioma stem cells by the Dirks group.

## Growth factors in combination

Each group whose publications were studied used different combinations of growth factors. For example, the Dirks group used a combination of three growth factors: EGF, bFGF, and leukemia inhibitory factor, as well as two other factors as the Weissman group did. The Rich group followed the Dirks method to culture glioma stem cells. However, *Carpenter* et al. only used EGF, bFGF, and leukemia inhibitory factors. The Weiss group only used EGF and bFGF with heparan sulfate, and the Fine group used only two growth factors (EGF and bFGF) in neurobasal media with bFGF and EGF (NBE) media (consisting of neurobasal media, N2, and B27 (0.5× each)). Therefore, differences in growth factor combinations may explain some of the discrepancies in their results.

## Concentration of growth factors

The concentrations of growth factors, especially EGF and bFGF, being used in the glioma stem cell media varied considerably among the published groups. The Dirks group used 20 ng/ml EGF, 20 ng/ml bFGF, and 10 ng/ml leukemia inhibitory factor. The Weiss group used 20 ng/ml EGF, 20 ng/ml bFGF, and 2 μg/ml heparan sulfate without leukemia inhibitory factor. However, the Fine group used media with 50 ng/ml EGF and 50 ng/ml bFGF, which doubled the concentration of those factors compared to the other groups. *Qian* et al. demonstrated that multipotent cortical stem cells have a fate choice that is environmentally directed and that neural stem cells exposed to low concentrations of bFGF (0.1 ng/ml) resulted in differentiation to neurons, while exposure to higher concentrations (1–10 ng/ml) resulted in differentiation to neurons and oligodendrocytes [64]. Therefore, differences in growth factor concentrations may generate different kinds of cells. This highlights the importance of standardizing growth factor concentrations for glioma stem cell culture as well as neural stem cell culture.

## The use of antibiotics

The prophylactic use of antibiotics in cell culture is commonly done to eliminate microbial contaminants and the most common contaminants are bacteria, yeast, fungi, and mycoplasma [65]. While mycoplasma may not affect the growth of cells, persistent infection can change the cells genetically and phenotypically [66, 67]. Generally, antibiotics in culture media are included at standard concentrations and are not believed to have toxic effects on the cells, but antibiotics still have the potential to affect cell function. The routine use of antibiotics in culture media is not recommended, because they may select for antibiotic-resistant strains of microorganisms [68]. *Cohen* et al. had suggested that the routine use of antibiotics in embryonic stem cell (ESC) cultures be avoided, as it might reduce the efficiency of the culture system [69]. Therefore, the use of antibiotics in glioma stem cell culture medium is not suggested. The use of antibiotics may interfere with cells in culture, resulting in unmatched culture conditions and questionable results.

## Conclusion

There is a critical need to review the culturing methods, the media, and components used in glioma stem cell culture. In this review, we summarize the most commonly used culture media and their components. We also emphasize the function of each component used in the culture media. In addition, we highlight the necessity of a standardized glioma stem cell culturing system. We propose that a standard glioma stem cell culture media should include neurobasal medium with N2 (Gibco), EGF, bFGF, leukemia inhibitory factor, and heparin to avoid inconsistencies in glioma stem cell research due to technical culture issues.

To avoid discrepancies in glioma stem cell-related research, a standardized glioma stem cell culture procedure is essential. We recommend neurobasal medium as a basal medium instead of DMEM/F-12. The reasons for this recommendation are as follows: Firstly, the neurobasal medium is commercially available, uniform, and ready to be used. Therefore, it can reduce bias due to user variability in making the medium. Secondly, the components in the neurobasal medium are similar to those in DMEM/F-12, even though there are reductions in NaCl, cysteine/cystine, and glutamine. Lastly, the neurobasal media also contains the antioxidants vitamin E, glutathione, pyruvate, catalase, and superoxide dismutase, which help improve glioma cell survival. The addition of neuronal survival factor-1 and *N*-acetylcysteine is not needed in the culture medium.

In addition to the basal medium, supplements are also important components. The supplement we recommend is Gibco N2, and we do not recommend the addition of B27. Since there are overlapping components in N2 and B27, the overlapped components may affect the results of any downstream assays. In addition, the retinyl acetate and triiodo-l-thyronine in B27 have been reported to promote the differentiation of various precursor cells.

To maintain the long-term growth of glioma stem cells, the growth factor cocktail of EGF (20 ng/ml), bFGF (20 ng/ml), leukemia inhibitory factor, and heparin is also recommended for glioma stem cell culture. EGF can keep EGF-responsive glioma stem cells in suspension and bFGF can promote the long-term growth of glioma stem cells [24]. bFGF is unstable in a culture medium; therefore, heparin (5 μg/mL) should be included in the media to stabilize bFGF and prevent its degradation [54]. The fundamental differences between the growth requirements for glioma stem cells and neural stem cells have already been reported by the Weiss group [36]. Glioma stem cells are exogenous mitogen-independent cells while neural stem cells are dependent upon mitogens and require exogenous mitogens to proliferate. The addition of EGF and bFGF to the culture medium may increase the survival and proliferation of glioma stem cells and can alter their phenotypic potential toward that of neurons, astrocytes, and oligodendrocytes after differentiation in vitro. Importantly, glioma stem cells can initiate highly invasive tumors which resemble human glioblastomas when the glioma stem cells are transplanted into immunocompromised mice [36]. On the contrary, the neural stem cells could not form these tumors in mice.

*Carpenter* et al. noted that bFGF-responsive neural stem cells could not grow for an extended period of time, but this could be overcome by the addition of leukemia inhibitory factor (10 mg/m) to the medium [50]. In addition, leukemia inhibitory factor could maintain the stemness of glioma stem cells, and EGF, bFGF, and leukemia inhibitory factor together enable the expansion of neural stem cells in culture and allow them to retain their multipotency for at least 1 year in vitro [50]. Therefore, the use of leukemia inhibitory factor in the standard culture media is also recommended.

In addition to the characteristic differences between glioma stem cells and neural stem cells, they also have different sensitivities to chemotherapy and radiotherapy. *Gong* et al. reported that temozolomide decreased neural stem cell viability while minimally affecting glioma stem cells. In addition, temozolomide could induce cell death in neural stem cells but not in glioma stem cells [70, 71]. The reduction or death of neural stem cells may, at least partially, explain the cognitive impairments seen in glioblastoma patients after treatment with temozolomide. Moreover, the effects of radiotherapy on neural stem cells are also different from its effects on glioma stem cells. Radiotherapy induces acute apoptosis in dividing cells and reduces the pool of mitotic neural stem cells, mainly the activated neural stem cells and the neural progenitor cells [72]. Glioma stem cells are, however, resistant to radiation therapy [15]. To further study the mechanism of glioma stem cell resistance to chemoradiotherapy, more researches are needed in this field and standardized media and procedures are crucial.

In summary, there is a necessity for standardization of glioma stem cell culture to prevent biased results caused by the medium components and we propose a standard glioma stem cell culture media, which should include neurobasal medium with N2 (Gibco), EGF, bFGF, leukemia inhibitory factor, and heparin to avoid discrepancies in the results of glioma stem cell studies due to technical culture issues.

## Abbreviations

bFGF: Basic fibroblast growth factor
CNS: Central nervous system
DMEM: Dulbecco’s modified Eagle’s medium
EGF: Epidermal growth factor
ESC: Embryonic stem cells
GBM: Glioblastoma multiforme
HEPES: N-2-hydroxyethylpiperazine-N-2 ethane sulfonic acid
Hormone mix: Hormone and salt mixture
NAC: *N*-acetylcysteine
NBE: Neurobasal media with bFGF and EGF
NSCs: Neural stem cells
NSF-1: Neuronal survival factor-1
ROS: Reactive oxygen species
SFM: Serum-free medium
